# 4-[(*E*)-2-(2,4-Dichloro­benzyl­idene)hydrazin-1-yl]quinolin-1-ium chloride monohydrate

**DOI:** 10.1107/S1600536812012962

**Published:** 2012-03-31

**Authors:** Solange M. S. V. Wardell, Edward R. T. Tiekink, James L. Wardell, Marcelle de Lima Ferreira, Marcus V. N. de Souza

**Affiliations:** a CHEMSOL, 1 Harcourt Road, Aberdeen AB15 5NY, Scotland; b Department of Chemistry, University of Malaya, 50603 Kuala Lumpur, Malaysia; c Centro de Desenvolvimento Tecnológico em Saúde (CDTS), Fundação Oswaldo Cruz (FIOCRUZ), Casa Amarela, Campus de Manguinhos, Av. Brasil 4365, 21040-900 Rio de Janeiro, RJ, Brazil; d Instituto de Tecnologia em Fármacos–Farmanguinhos, FioCruz–Fundação Oswaldo Cruz, R. Sizenando Nabuco, 100, Manguinhos, 21041-250 Rio de Janeiro, RJ, Brazil

## Abstract

In the title hydrated salt, C_16_H_12_Cl_2_N_3_^+^·Cl^−^·H_2_O, there is a small twist in the cation as seen in the torsion angle linking the benzene ring to the rest of the mol­ecule [171.96 (17)°]. In the crystal, the quinolinium H atom forms a hydrogen bond to the lattice water mol­ecule, which also forms hydrogen bonds to two Cl^−^ anions. Each Cl^−^ ion also accepts a hydrogen bond from the hydrazine H atom. The three-dimensional architecture is also stabilized by π–π inter­actions between centrosymmetrically related quinoline residues [centroid–centroid distance = 3.5574 (11) Å].

## Related literature

For the biological activity, including anti-tubercular and anti-tumour activity, of compounds containing the quinolinyl nucleus, see: de Souza *et al.* (2009 ▶), Candea *et al.* (2009 ▶); Montenegro *et al.* (2011 ▶, 2012 ▶). For related structures, see: Howie *et al.* (2010 ▶); de Souza *et al.* (2010 ▶).
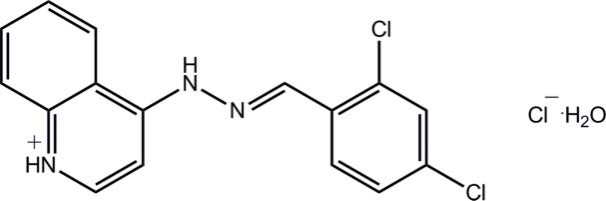


## Experimental

### 

#### Crystal data

C_16_H_12_Cl_2_N_3_^+^·Cl^−^·H_2_O
*M* *_r_* = 370.65
Triclinic, 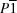

*a* = 7.6815 (2) Å
*b* = 9.7491 (3) Å
*c* = 10.8418 (3) Å
α = 87.831 (2)°
β = 87.171 (2)°
γ = 87.146 (2)°
*V* = 809.41 (4) Å^3^
*Z* = 2
Mo *K*α radiation
μ = 0.57 mm^−1^
*T* = 120 K
0.10 × 0.09 × 0.08 mm

#### Data collection

Bruker–Nonius Roper CCD camera on a κ-goniostat diffractometer
Absorption correction: multi-scan (*SADABS*; Sheldrick, 2007 ▶) *T*_min_ = 0.666, *T*_max_ = 0.746
16815 measured reflections
3701 independent reflections
3016 reflections with *I* > 2σ(*I*)
*R*_int_ = 0.056

#### Refinement

*R*[*F* ^2^ > 2σ(*F* ^2^)] = 0.041
*wR*(*F* ^2^) = 0.103
*S* = 1.06
3701 reflections
220 parameters
5 restraints
H atoms treated by a mixture of independent and constrained refinement
Δρ_max_ = 0.36 e Å^−3^
Δρ_min_ = −0.34 e Å^−3^

### 

Data collection: *COLLECT* (Hooft, 1998 ▶); cell refinement: *DENZO* (Otwinowski & Minor, 1997 ▶) and *COLLECT*; data reduction: *DENZO* and *COLLECT*; program(s) used to solve structure: *SHELXS97* (Sheldrick, 2008 ▶); program(s) used to refine structure: *SHELXL97* (Sheldrick, 2008 ▶); molecular graphics: *ORTEP-3* (Farrugia, 1997 ▶) and *DIAMOND* (Brandenburg, 2006 ▶); software used to prepare material for publication: *publCIF* (Westrip, 2010 ▶).

## Supplementary Material

Crystal structure: contains datablock(s) global, I. DOI: 10.1107/S1600536812012962/xu5495sup1.cif

Structure factors: contains datablock(s) I. DOI: 10.1107/S1600536812012962/xu5495Isup2.hkl

Supplementary material file. DOI: 10.1107/S1600536812012962/xu5495Isup3.cml

Additional supplementary materials:  crystallographic information; 3D view; checkCIF report

## Figures and Tables

**Table 1:** Hydrogen-bond geometry (Å, °)

*D*—H⋯*A*	*D*—H	H⋯*A*	*D*⋯*A*	*D*—H⋯*A*
N1—H1*n*⋯O1*w*	0.88 (2)	1.80 (2)	2.673 (2)	170 (2)
O1*w*—H1*w*⋯Cl3^i^	0.84 (2)	2.32 (2)	3.1451 (18)	169 (3)
N2—H2*n*⋯Cl3	0.88 (1)	2.36 (1)	3.2175 (16)	166 (2)
O1*w*—H2*w*⋯Cl3^ii^	0.84 (2)	2.30 (2)	3.1295 (19)	173 (3)

Symmetry codes: (i) 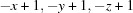; (ii) 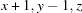.

**Fig. 1.**
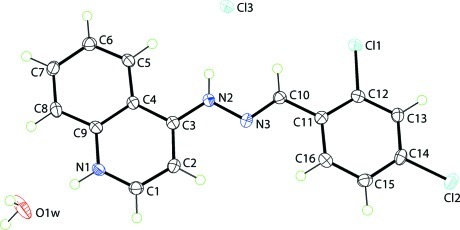
The molecular structure showing the atom-labelling scheme and displacement ellipsoids at the 50% probability level.

**Fig. 2.**
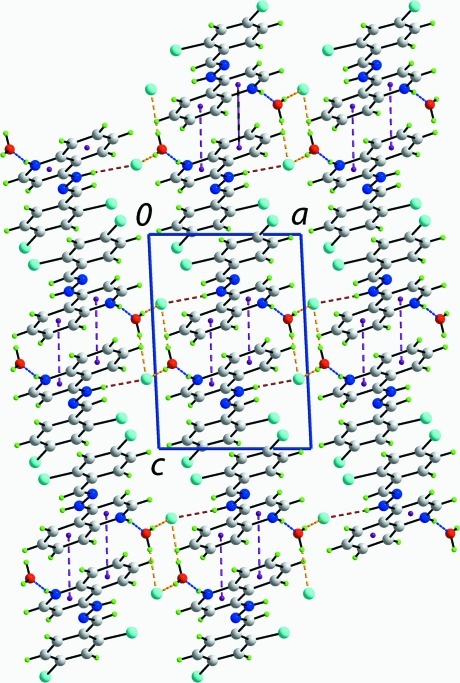
A view in projection down the b axis of the unit-cell contents of (I). The O—H···Cl, N—H···O, N—H···Cl and π···π interactions are shown as orange, blue, brown and purple dashed lines, respectively.

## supplementary crystallographic information

### Comment

A wide range of pharmacological activities have been noted for compounds containing the quinoline nucleus (de Souza *et al.*, 2009), including anti-tubercular (Candea *et al.*, 2009) and anti-tumour (Montenegro *et al.*, 2012) activities. Recently, we have focused attention on arylaldehyde 7-chloroquinoline-4-hydrazone derivatives (Candea *et al.*, 2009; Montenegro *et al.*, 2011). Complementing synthetic studies are crystallographic investigations of the these hydrazones (Howie *et al.*, 2010; de Souza *et al.*, 2010). In this connection, we now wish to report the crystal structure of the title hydrated salt, (I).

The asymmetric unit of (I), Fig. 1, comprises a 4-[(*E*)-2-[(2,4-dichlorophenyl)methylidene]hydrazin-1-yl]quinolin-1-ium cation, a chloride anion and a solvent water molecule. There is a small twist about the C10—C11 bond as seen in the value of the N3—C10—C11—C12 torsion angle, *i.e*. 171.96 (17)°. Nevertheless, the entire molecule is approximately planar with the r.m.s. deviation of all 24 non-hydrogen atoms being 0.072 Å. The maximum deviations from the least-squares plane are 0.148 (2) for the C14 atom and -0.130 (1) Å for the Cl1 atom. The conformation about the N3═C10 bond [1.286 (2) Å] is *E*.

There are a number of hydrogen-bonding interactions operating in the crystal structure of (I), Table 1. The pyridinium-H forms a hydrogen bond to the water molecule which links two chloride anions *via* O—H···Cl interactions. Through a centre of inversion, an eight-membered {···HOH···Cl}_2_ synthon is formed. Finally, the hydrazine-H atom forms a hydrogen bond to the chloride atom. The three-dimensional architecture is also stabilized by π—π interactions between centrosymmetrically related quinolinyl residues [centroid···centroid distance = 3.5574 (11) Å for symmetry operation: 1 - *x*, 1 - *y*, 1 - *z*], Fig. 2.

### Experimental

The compound was prepared from 7-chloro-4-quinolinylhydrazone with 2,5-dimethoxybenzaldehyde (Montenegro *et al.*, 2012). The crystals used in the structure determination were grown from an ethanol solution of the compound.

### Refinement

The C-bound H atoms were geometrically placed (C—H = 0.95 Å) and refined as riding with *U*_iso_(H) = 1.2*U*_eq_(C). The N-bound and O-bound H-atoms were located in a difference Fourier map and refined with a O—H = 0.84±0.01 Å [*U*_iso_(H) = 1.5*U*_eq_(O)] and N—H = 0.88±0.01 Å [*U*_iso_(H) = 1.2*U*_eq_(N)]. Owing to poor agreement, the (1 1 3) reflection was omitted from the final cycles of refinement.

### Crystal data

**Table:**

C_16_H_12_Cl_2_N_3_^+^·Cl^−^·H_2_O	*Z* = 2
*M**_r_* = 370.65	*F*(000) = 380
Triclinic, *P*1	*D*_x_ = 1.521 Mg m^−^^3^
Hall symbol: -P 1	Mo *K*α radiation, λ = 0.71073 Å
*a* = 7.6815 (2) Å	Cell parameters from 10943 reflections
*b* = 9.7491 (3) Å	θ = 2.9–27.5°
*c* = 10.8418 (3) Å	µ = 0.57 mm^−^^1^
α = 87.831 (2)°	*T* = 120 K
β = 87.171 (2)°	Block, colourless
γ = 87.146 (2)°	0.10 × 0.09 × 0.08 mm
*V* = 809.41 (4) Å^3^	

### Data collection

**Table:**

Bruker–Nonius Roper CCD camera on a κ-goniostat diffractometer	3701 independent reflections
Radiation source: Bruker–Nonius FR591 rotating anode	3016 reflections with *I* > 2σ(*I*)
Graphite monochromator	*R*_int_ = 0.056
Detector resolution: 9.091 pixels mm^-1^	θ_max_ = 27.5°, θ_min_ = 3.2°
φ and ω scans	*h* = −9→9
Absorption correction: multi-scan (*SADABS*; Sheldrick, 2007)	*k* = −12→12
*T*_min_ = 0.666, *T*_max_ = 0.746	*l* = −14→14
16815 measured reflections	

### Refinement

**Table:**

Refinement on *F*^2^	Primary atom site location: structure-invariant direct methods
Least-squares matrix: full	Secondary atom site location: difference Fourier map
*R*[*F*^2^ > 2σ(*F*^2^)] = 0.041	Hydrogen site location: inferred from neighbouring sites
*wR*(*F*^2^) = 0.103	H atoms treated by a mixture of independent and constrained refinement
*S* = 1.06	*w* = 1/[σ^2^(*F*_o_^2^) + (0.0455*P*)^2^ + 0.2111*P*] where *P* = (*F*_o_^2^ + 2*F*_c_^2^)/3
3701 reflections	(Δ/σ)_max_ = 0.001
220 parameters	Δρ_max_ = 0.36 e Å^−^^3^
5 restraints	Δρ_min_ = −0.34 e Å^−^^3^

### Special details

**Table:**

Geometry. All s.u.'s (except the s.u. in the dihedral angle between two l.s. planes) are estimated using the full covariance matrix. The cell s.u.'s are taken into account individually in the estimation of s.u.'s in distances, angles and torsion angles; correlations between s.u.'s in cell parameters are only used when they are defined by crystal symmetry. An approximate (isotropic) treatment of cell s.u.'s is used for estimating s.u.'s involving l.s. planes.
Refinement. Refinement of *F*^2^ against ALL reflections. The weighted *R*-factor *wR* and goodness of fit *S* are based on *F*^2^, conventional *R*-factors *R* are based on *F*, with *F* set to zero for negative *F*^2^. The threshold expression of *F*^2^ > 2σ(*F*^2^) is used only for calculating *R*-factors(gt) *etc*. and is not relevant to the choice of reflections for refinement. *R*-factors based on *F*^2^ are statistically about twice as large as those based on *F*, and *R*- factors based on ALL data will be even larger.

### Fractional atomic coordinates and isotropic or equivalent isotropic displacement parameters (Å^2^)

**Table:**

	*x*	*y*	*z*	*U*_iso_*/*U*_eq_	
Cl1	0.23598 (6)	1.20701 (5)	0.14241 (5)	0.02343 (14)	
Cl2	0.81838 (6)	1.41676 (5)	−0.06262 (5)	0.02564 (14)	
N1	0.7250 (2)	0.35248 (17)	0.32132 (15)	0.0184 (3)	
H1n	0.785 (2)	0.2747 (14)	0.337 (2)	0.022*	
N2	0.4580 (2)	0.72610 (16)	0.26643 (15)	0.0176 (3)	
H2n	0.3464 (14)	0.740 (2)	0.2871 (19)	0.021*	
N3	0.5488 (2)	0.83194 (16)	0.21124 (14)	0.0177 (3)	
C1	0.8043 (3)	0.4576 (2)	0.26455 (18)	0.0207 (4)	
H1	0.9235	0.4453	0.2378	0.025*	
C2	0.7194 (2)	0.5834 (2)	0.24339 (17)	0.0196 (4)	
H2	0.7794	0.6566	0.2029	0.024*	
C3	0.5435 (2)	0.60214 (19)	0.28223 (16)	0.0161 (4)	
C4	0.4548 (2)	0.48914 (19)	0.34195 (16)	0.0157 (4)	
C5	0.2776 (2)	0.4957 (2)	0.38498 (17)	0.0186 (4)	
H5	0.2080	0.5778	0.3719	0.022*	
C6	0.2056 (3)	0.3845 (2)	0.44529 (18)	0.0207 (4)	
H6	0.0868	0.3907	0.4742	0.025*	
C7	0.3057 (3)	0.2609 (2)	0.46494 (18)	0.0218 (4)	
H7	0.2544	0.1849	0.5074	0.026*	
C8	0.4769 (3)	0.2506 (2)	0.42284 (18)	0.0202 (4)	
H8	0.5441	0.1672	0.4353	0.024*	
C9	0.5527 (2)	0.36373 (19)	0.36120 (16)	0.0167 (4)	
C10	0.4620 (2)	0.94746 (19)	0.19875 (17)	0.0176 (4)	
H10	0.3440	0.9576	0.2288	0.021*	
C11	0.5500 (2)	1.06322 (19)	0.13685 (17)	0.0171 (4)	
C12	0.4584 (2)	1.18619 (19)	0.10639 (17)	0.0174 (4)	
C13	0.5395 (2)	1.2951 (2)	0.04510 (17)	0.0190 (4)	
H13	0.4754	1.3783	0.0258	0.023*	
C14	0.7152 (3)	1.27973 (19)	0.01280 (17)	0.0193 (4)	
C15	0.8113 (2)	1.1589 (2)	0.04001 (18)	0.0210 (4)	
H15	0.9317	1.1491	0.0157	0.025*	
C16	0.7286 (3)	1.0533 (2)	0.10297 (18)	0.0207 (4)	
H16	0.7945	0.9715	0.1241	0.025*	
Cl3	0.05983 (6)	0.83146 (5)	0.32453 (5)	0.02497 (14)	
O1w	0.8874 (3)	0.11709 (18)	0.39542 (16)	0.0445 (5)	
H1w	0.908 (4)	0.119 (3)	0.4704 (12)	0.067*	
H2w	0.933 (4)	0.0432 (19)	0.370 (3)	0.067*	

### Atomic displacement parameters (Å^2^)

**Table:**

	*U*^11^	*U*^22^	*U*^33^	*U*^12^	*U*^13^	*U*^23^
Cl1	0.0168 (2)	0.0208 (3)	0.0321 (3)	0.00144 (18)	0.00122 (19)	0.0017 (2)
Cl2	0.0283 (3)	0.0229 (3)	0.0258 (3)	−0.0093 (2)	0.0037 (2)	0.0014 (2)
N1	0.0188 (8)	0.0192 (8)	0.0164 (8)	0.0052 (7)	0.0006 (6)	0.0008 (7)
N2	0.0171 (8)	0.0150 (8)	0.0206 (8)	0.0000 (6)	−0.0001 (6)	0.0007 (6)
N3	0.0196 (8)	0.0164 (8)	0.0173 (8)	−0.0033 (6)	−0.0013 (6)	0.0015 (6)
C1	0.0190 (9)	0.0238 (10)	0.0187 (10)	0.0020 (8)	0.0007 (8)	0.0009 (8)
C2	0.0209 (9)	0.0189 (10)	0.0187 (10)	−0.0016 (8)	0.0001 (8)	0.0026 (8)
C3	0.0198 (9)	0.0174 (9)	0.0112 (8)	0.0004 (7)	−0.0021 (7)	−0.0019 (7)
C4	0.0186 (9)	0.0162 (9)	0.0125 (9)	0.0005 (7)	−0.0026 (7)	−0.0007 (7)
C5	0.0201 (9)	0.0176 (10)	0.0177 (9)	0.0032 (8)	−0.0018 (8)	−0.0009 (7)
C6	0.0208 (10)	0.0207 (10)	0.0205 (10)	−0.0015 (8)	0.0008 (8)	−0.0019 (8)
C7	0.0257 (10)	0.0189 (10)	0.0204 (10)	−0.0030 (8)	0.0020 (8)	0.0022 (8)
C8	0.0270 (10)	0.0154 (9)	0.0180 (10)	0.0022 (8)	−0.0031 (8)	0.0011 (7)
C9	0.0190 (9)	0.0179 (10)	0.0131 (9)	0.0020 (8)	−0.0017 (7)	−0.0022 (7)
C10	0.0176 (9)	0.0182 (10)	0.0169 (9)	−0.0005 (8)	−0.0001 (7)	−0.0003 (7)
C11	0.0187 (9)	0.0180 (10)	0.0150 (9)	−0.0025 (7)	−0.0009 (7)	−0.0021 (7)
C12	0.0163 (9)	0.0187 (10)	0.0175 (9)	−0.0004 (7)	−0.0019 (7)	−0.0034 (7)
C13	0.0229 (10)	0.0162 (9)	0.0179 (10)	−0.0009 (8)	−0.0013 (8)	0.0010 (7)
C14	0.0247 (10)	0.0169 (10)	0.0172 (9)	−0.0078 (8)	−0.0023 (8)	−0.0005 (7)
C15	0.0173 (9)	0.0230 (10)	0.0229 (10)	−0.0020 (8)	0.0008 (8)	−0.0031 (8)
C16	0.0210 (10)	0.0186 (10)	0.0225 (10)	0.0020 (8)	−0.0024 (8)	−0.0016 (8)
Cl3	0.0219 (3)	0.0205 (3)	0.0312 (3)	0.00329 (19)	0.0060 (2)	0.0001 (2)
O1w	0.0646 (12)	0.0317 (9)	0.0356 (10)	0.0289 (9)	−0.0141 (9)	−0.0066 (8)

### Geometric parameters (Å, º)

**Table:**

Cl1—C12	1.7374 (19)	C6—H6	0.9500
Cl2—C14	1.7435 (19)	C7—C8	1.371 (3)
N1—C1	1.334 (3)	C7—H7	0.9500
N1—C9	1.372 (2)	C8—C9	1.404 (3)
N1—H1n	0.884 (9)	C8—H8	0.9500
N2—C3	1.355 (2)	C10—C11	1.468 (3)
N2—N3	1.376 (2)	C10—H10	0.9500
N2—H2n	0.881 (9)	C11—C12	1.396 (3)
N3—C10	1.286 (2)	C11—C16	1.402 (3)
C1—C2	1.377 (3)	C12—C13	1.388 (3)
C1—H1	0.9500	C13—C14	1.380 (3)
C2—C3	1.401 (3)	C13—H13	0.9500
C2—H2	0.9500	C14—C15	1.389 (3)
C3—C4	1.440 (3)	C15—C16	1.377 (3)
C4—C9	1.418 (2)	C15—H15	0.9500
C4—C5	1.416 (3)	C16—H16	0.9500
C5—C6	1.371 (3)	O1w—H1w	0.836 (10)
C5—H5	0.9500	O1w—H2w	0.834 (10)
C6—C7	1.412 (3)		
			
C1—N1—C9	121.52 (16)	C7—C8—C9	119.77 (17)
C1—N1—H1n	119.8 (14)	C7—C8—H8	120.1
C9—N1—H1n	118.6 (14)	C9—C8—H8	120.1
C3—N2—N3	118.12 (16)	N1—C9—C8	119.09 (16)
C3—N2—H2n	122.5 (14)	N1—C9—C4	119.98 (17)
N3—N2—H2n	119.3 (14)	C8—C9—C4	120.93 (17)
C10—N3—N2	115.69 (16)	N3—C10—C11	118.31 (17)
N1—C1—C2	122.28 (18)	N3—C10—H10	120.8
N1—C1—H1	118.9	C11—C10—H10	120.8
C2—C1—H1	118.9	C12—C11—C16	117.33 (17)
C1—C2—C3	119.17 (18)	C12—C11—C10	121.41 (17)
C1—C2—H2	120.4	C16—C11—C10	121.24 (17)
C3—C2—H2	120.4	C13—C12—C11	121.87 (17)
N2—C3—C2	120.53 (17)	C13—C12—Cl1	117.49 (14)
N2—C3—C4	120.06 (17)	C11—C12—Cl1	120.64 (15)
C2—C3—C4	119.39 (17)	C14—C13—C12	118.55 (18)
C9—C4—C5	117.85 (17)	C14—C13—H13	120.7
C9—C4—C3	117.65 (17)	C12—C13—H13	120.7
C5—C4—C3	124.49 (17)	C13—C14—C15	121.63 (18)
C6—C5—C4	120.52 (17)	C13—C14—Cl2	118.79 (15)
C6—C5—H5	119.7	C15—C14—Cl2	119.58 (15)
C4—C5—H5	119.7	C16—C15—C14	118.69 (18)
C5—C6—C7	120.85 (18)	C16—C15—H15	120.7
C5—C6—H6	119.6	C14—C15—H15	120.7
C7—C6—H6	119.6	C15—C16—C11	121.90 (18)
C8—C7—C6	120.07 (18)	C15—C16—H16	119.0
C8—C7—H7	120.0	C11—C16—H16	119.0
C6—C7—H7	120.0	H1w—O1w—H2w	107 (3)
			
C3—N2—N3—C10	−179.79 (16)	C5—C4—C9—N1	−179.89 (16)
C9—N1—C1—C2	0.5 (3)	C3—C4—C9—N1	−1.2 (3)
N1—C1—C2—C3	−0.2 (3)	C5—C4—C9—C8	−1.1 (3)
N3—N2—C3—C2	0.1 (3)	C3—C4—C9—C8	177.65 (16)
N3—N2—C3—C4	178.67 (15)	N2—N3—C10—C11	−178.02 (15)
C1—C2—C3—N2	177.78 (17)	N3—C10—C11—C12	171.96 (17)
C1—C2—C3—C4	−0.8 (3)	N3—C10—C11—C16	−6.3 (3)
N2—C3—C4—C9	−177.12 (16)	C16—C11—C12—C13	0.0 (3)
C2—C3—C4—C9	1.5 (3)	C10—C11—C12—C13	−178.37 (17)
N2—C3—C4—C5	1.5 (3)	C16—C11—C12—Cl1	178.98 (14)
C2—C3—C4—C5	−179.93 (17)	C10—C11—C12—Cl1	0.6 (3)
C9—C4—C5—C6	1.2 (3)	C11—C12—C13—C14	0.6 (3)
C3—C4—C5—C6	−177.37 (17)	Cl1—C12—C13—C14	−178.43 (14)
C4—C5—C6—C7	−0.5 (3)	C12—C13—C14—C15	0.1 (3)
C5—C6—C7—C8	−0.4 (3)	C12—C13—C14—Cl2	−179.28 (14)
C6—C7—C8—C9	0.6 (3)	C13—C14—C15—C16	−1.3 (3)
C1—N1—C9—C8	−178.63 (17)	Cl2—C14—C15—C16	178.04 (15)
C1—N1—C9—C4	0.2 (3)	C14—C15—C16—C11	1.9 (3)
C7—C8—C9—N1	178.98 (17)	C12—C11—C16—C15	−1.3 (3)
C7—C8—C9—C4	0.1 (3)	C10—C11—C16—C15	177.08 (17)

### Hydrogen-bond geometry (Å, º)

**Table:**

*D*—H···*A*	*D*—H	H···*A*	*D*···*A*	*D*—H···*A*
N1—H1*n*···O1*w*	0.88 (2)	1.80 (2)	2.673 (2)	170 (2)
O1*w*—H1*w*···Cl3^i^	0.84 (2)	2.32 (2)	3.1451 (18)	169 (3)
N2—H2*n*···Cl3	0.88 (1)	2.36 (1)	3.2175 (16)	166 (2)
O1*w*—H2*w*···Cl3^ii^	0.84 (2)	2.30 (2)	3.1295 (19)	173 (3)

Symmetry codes: (i) −*x*+1, −*y*+1, −*z*+1; (ii) *x*+1, *y*−1, *z*.
